# Colocalization of 14-3-3 Proteins with SOD1 in Lewy Body-Like Hyaline Inclusions in Familial Amyotrophic Lateral Sclerosis Cases and the Animal Model

**DOI:** 10.1371/journal.pone.0020427

**Published:** 2011-05-31

**Authors:** Yoko Okamoto, Yoshitomo Shirakashi, Masafumi Ihara, Makoto Urushitani, Miki Oono, Yasuhiro Kawamoto, Hirofumi Yamashita, Shun Shimohama, Shinsuke Kato, Asao Hirano, Hidekazu Tomimoto, Hidefumi Ito, Ryosuke Takahashi

**Affiliations:** 1 Department of Neurology, Kyoto University Graduate School of Medicine, Kyoto, Japan; 2 Molecular Neuroscience Research Center, Shiga University of Medical Science, Shiga, Japan; 3 Department of Neurology, Sapporo Medical University School of Medicine, Hokkaido, Japan; 4 Department of Neuropathology, Institute of Neurological Sciences, Faculty of Medicine, Tottori University, Tottori, Japan; 5 Division of Neuropathology, Department of Pathology, Montefiore Medical Center, New York, New York, United States of America; 6 Department of Neurology, Mie University Graduate School of Medicine, Mie, Japan; Brigham and Women's Hospital, Harvard Medical School, United States of America

## Abstract

**Background and Purpose:**

Cu/Zn superoxide dismutase (SOD1) is a major component of Lewy body-like hyaline inclusion (LBHI) found in the postmortem tissue of SOD1-linked familial amyotrophic lateral sclerosis (FALS) patients. In our recent studies, 14-3-3 proteins have been found in the ubiquitinated inclusions inside the anterior horn cells of spinal cords with sporadic amyotrophic lateral sclerosis (ALS). To further investigate the role of 14-3-3 proteins in ALS, we performed immunohistochemical analysis of 14-3-3 proteins and compared their distributions with those of SOD1 in FALS patients and SOD1-overexpressing mice.

**Methods:**

We examined the postmortem brains and the spinal cords of three FALS cases (A4V SOD1 mutant). Transgenic mice expressing the G93A mutant human SOD1 (mutant SOD1-Tg mice), transgenic mice expressing the wild-type human SOD1 (wild-type SOD1-Tg mice), and non-Tg wild-type mice were also subjected to the immunohistochemical analysis.

**Results:**

In all the FALS patients, LBHIs were observed in the cytoplasm of the anterior horn cells, and these inclusions were immunopositive intensely for pan 14-3-3, 14-3-3β, and 14-3-3γ. In the mutant SOD1-Tg mice, a high degree of immunoreactivity for misfolded SOD1 (C4F6) was observed in the cytoplasm, with an even greater degree of immunoreactivity present in the cytoplasmic aggregates of the anterior horn cells in the lumbar spinal cord. Furthermore, we have found increased 14-3-3β and 14-3-3γ immunoreactivities in the mutant SOD1-Tg mice. Double immunofluorescent staining showed that C4F6 and 14-3-3 proteins were partially co-localized in the spinal cord with FALS and the mutant SOD1-Tg mice. In comparison, the wild-type SOD1-Tg and non-Tg wild-type mice showed no or faint immunoreactivity for C4F6 and 14-3-3 proteins (pan 14-3-3, 14-3-3β, and 14-3-3γ) in any neuronal compartments.

**Discussion:**

These results suggest that 14-3-3 proteins may be associated with the formation of SOD1-containing inclusions, in FALS patients and the mutant SOD1-Tg mice.

## Introduction

Amyotrophic lateral sclerosis (ALS) is a fatal, progressive neurodegenerative disease characterized by the degeneration of motor neurons in the motor cortex, brainstem and spinal cord. The vast majority of ALS patients are sporadic, and approximately 5–10% of ALS cases are familial ALS (FALS) [1]. Among the FALS patients, approximately 20% are linked to mutations in the antioxidant enzyme Cu/Zn superoxide dismutase (SOD1) [2]. Mutant SOD1 proteins aggregate and form Lewy body-like hyaline inclusions (LBHIs) in the anterior horn cells of the spinal cord [3].

Transgenic mice carrying several copies of human mutant SOD1 genes show ALS-like symptoms such as progressive motor disturbances and neurogenic amyotrophy, and develop a pathology resembling ALS [4]. In brief, these Tg mice demonstrate atrophy of the motor neuronal system, vacuolar degeneration of the motor neurons, and ubiquitinated neuronal hyaline inclusions which contain SOD1 in their cell bodies and swollen processes [5].

SOD1 is a major constituent of LBHIs linked to FALS, and these LBHIs contain ubiquitin [6], phosphorylated neurofilaments [7], and a copper chaperone for superoxide dismutase [8].

The 14-3-3 proteins, a family of protein chaperones, are abundant in the brain, comprising approximately 1% of the total brain protein [9]. 14-3-3 proteins consist of seven different isoforms, named with Greek letters (β, ε, γ, η, θ, σ, and ζ). Each isoform forms homo- or hetero-dimers. 14-3-3 dimers can simultaneously bind two ligands, modulate different signaling molecules and participate in cell cycle control, cell adhesion, neuronal plasticity as well as various intracellular signal transduction pathways [10]. 14-3-3 proteins seem to control the subcellular localization of proteins and to function as adaptor molecules, stimulating protein-protein interactions. The regulation of this interaction usually involves the phosphorylation of the interacting proteins [11].

In our recent studies, several types of 14-3-3 proteins such as 14-3-3β, 14-3-3γ, 14-3-3ζ, 14-3-3θ, or 14-3-3ε have been found in the ubiquitinated inclusions of anterior horn cells from patients with sporadic ALS [12]. 14-3-3 mRNA was also demonstrated to be upregulated in the spinal cords with sporadic ALS [13].

However, the association of 14-3-3 proteins with FALS remains unknown. In this study, to investigate the role of 14-3-3 proteins and SOD1 in the pathogenesis of FALS, we performed immunohistochemical staining for 14-3-3 proteins and SOD1 in formalin-fixed, paraffin-embedded sections from patients with FALS. Transgenic mice which overexpress mutant human SOD1, transgenic mice which overexpress wild type human SOD1, and non-transgenic wild-type mice were also subjected to immunohistochemical analysis.

## Methods

### Ethics Statement

The protocols for genetic analysis and neuropathological procedures were approved by and performed under the guidelines of our institutional ethics committee. Informed consent was obtained from all individuals or their guardians before the analysis. The animal study was carried out in strict accordance with the guidelines for animal experimentation from the Animal Research Committee of our institution. The protocol was approved by the Animal Research Committee, Kyoto University (Permit Number: MedKyo10202).

### Human FALS cases

We analyzed three cases of FALS (A4V SOD1 mutant). The clinicopathological backgrounds of these FALS cases have been previously reported [14]. These patients were members of the American “C” family. The three patients were males, and their ages at death were 39, 46 and 66 years. They were pathologically consistent with FALS with posterior column involvement [15].

### Transgenic mice expressing G93A mutant human SOD1 and wild type human SOD1

We used transgenic mice expressing the G93A mutant human SOD1 gene (mutant SOD1-Tg mice) [B6SJL-TgN (SOD1-G93A) 1Gur] and wild-type human SOD1 gene (wild-type SOD1-Tg mice) [B6SJL-Tg (SOD1) 2Gur/J], which were originally obtained from the Jackson Laboratory [16]. The mutant SOD1-Tg mice develop signs of hind limb weakness at the age of 3 to 4 months. At the age of 5 to 6 months, they are not able to forage for food and water and then die. The wild-type SOD1-Tg mice show no motor symptoms [17]. We analyzed four-month-old mutant SOD1-Tg and wild-type SOD1-Tg mice (n = 4, each).

### Human tissues

Human tissue blocks obtained from the different levels of spinal cords of FALS cases were embedded in paraffin. The blocks were sectioned with a microtome at 6 µm thickness for routine and immunohistochemical staining. Routine histological assessment was carried out with hematoxylin and eosin (H&E).

H&E-stained sections with LBHIs were photographed, decolorized with 70% ethanol and pretreated with 0.3% H_2_O_2_ in 0.1 mol/L phosphate-buffered saline (PBS) for 30 min at room temperature to inhibit any endogenous peroxidase activity. After washing with 0.1 mol/L PBS, these sections were blocked with 0.1 mol/L PBS plus 3% skim milk for 2 hours at room temperature. Then, the specimens were used for immunohistochemistry; this involved sequential incubation with primary antibody, appropriate biotinylated secondary antibody (Vector Laboratories, diluted 1∶200), and avidin-biotin-peroxidase complex (ABC; Vector Laboratories, 1∶200) in 0.1 mol/L PBS containing 0.3% Triton X-100 (PBST, pH 7.4). The sections were rinsed with PBST for 15 min between each step and finally visualized with 0.01% diaminobenzidinetetrahydrochloride and 0.005% H_2_O_2_ in 50 mmol/L Tris-HCl (pH 7.6).

### Animal tissues

Mice were deeply anesthetized with sodium pentobarbital and were perfused transcardially with 0.01 mol/L PBS and then with a fixative containing 4% paraformaldehyde (PFA) and 0.2% picric acid in 0.1 mol/L phosphate buffer (PB, pH 7.4). Then, the brains and the spinal cords were removed. The tissues were post-fixed for 24 hours in 4% PFA and stored in 20% sucrose in 0.1 mol/L PB (pH 7.4). Serial lumbar spinal sections were cut into 20 µm thick sections on a cryostat, and immunohistochemical analysis was performed in the same way as human tissues described above.

### Primary antibodies

As primary antibodies, we used anti-SOD1, anti-misfolded SOD1 (C4F6) [18], and anti- pan and isoform-specific 14-3-3 protein antibodies. The primary antibodies used were listed in Table 1.

**Table 1:** Primary antibodies.

Primary antibody				Company	Dilution
C4F6	mouse monoclonal			Reference [18]	1∶1000
SOD1	goat polyclonal	C-17	SC-8637	Santa Cruz Biotechnology	1∶1000
pan 14-3-3	mouse monoclonal	H-8	SC-1657	Santa Cruz Biotechnology	1∶1000
14-3-3β	rabbit polyclonal	C-20	SC-628	Santa Cruz Biotechnology	1∶2000
14-3-3γ	rabbit polyclonal	C-16	SC-731	Santa Cruz Biotechnology	1∶2000
14-3-3ε	rabbit polyclonal	T-16	SC-1020	Santa Cruz Biotechnology	1∶400
14-3-3η	goat polyclonal	E-12	SC-17287	Santa Cruz Biotechnology	1∶400
14-3-3θ	rabbit polyclonal	C-17	SC-732	Santa Cruz Biotechnology	1∶2000
14-3-3σ	goat polyclonal	C-18	SC-7683	Santa Cruz Biotechnology	1∶400
14-3-3σ	goat polyclonal	N-14	SC-7681	Santa Cruz Biotechnology	1∶400
14-3-3ζ	rabbit polyclonal	C-16	SC-1019	Santa Cruz Biotechnology	1∶2000

### Double labeling immunohistochemistry

To investigate the relationship between SOD1 and 14-3-3 proteins, spinal cord sections of the FALS cases were incubated with primary antibodies against SOD1 and pan 14-3-3, followed by incubation with FITC- or rhodamine-labeled appropriate secondary antibodies. For mouse tissues, anti-14-3-3 (β or γ) and C4F6-DyLight 488 antibodies were used. C4F6 was labeled with DyLight Fluor 488 using a commercially available kit (DyLight Microscale Antibody Labeling Kits, Thermo Scientific).

## Results

### 14-3-3 immunoreactivity in patients with FALS

**Figure 1:**
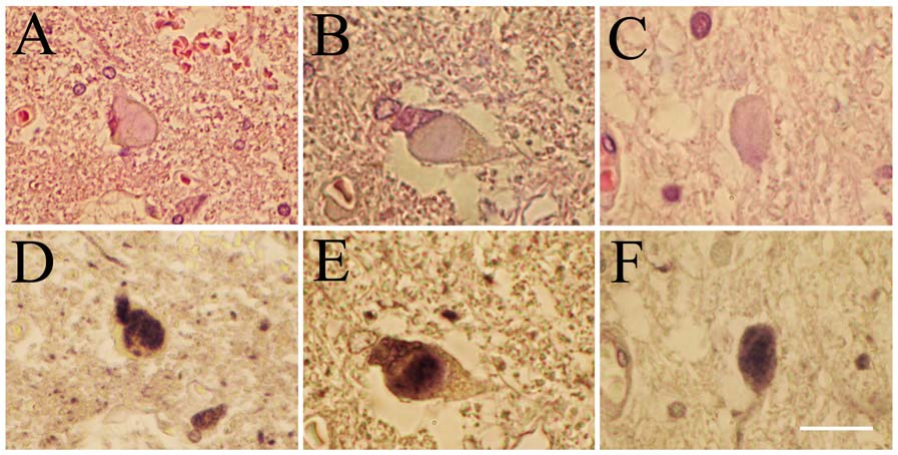
LBHIs immunopositive for 14-3-3 proteins in FALS patients. A, B, and C are the same sections as D, E, and F, respectively. The upper panels (A–C) are stained with H&E, and the lower panels (D–F) are immunostained with the anti-pan 14-3-3 antibody. LBHIs observed on H&E in the anterior horn cells are intensely immunopositive for pan 14-3-3. Bar indicates 100 µm in (A, D), and 50 µm in (B, C, E, F).

**Figure 2:**
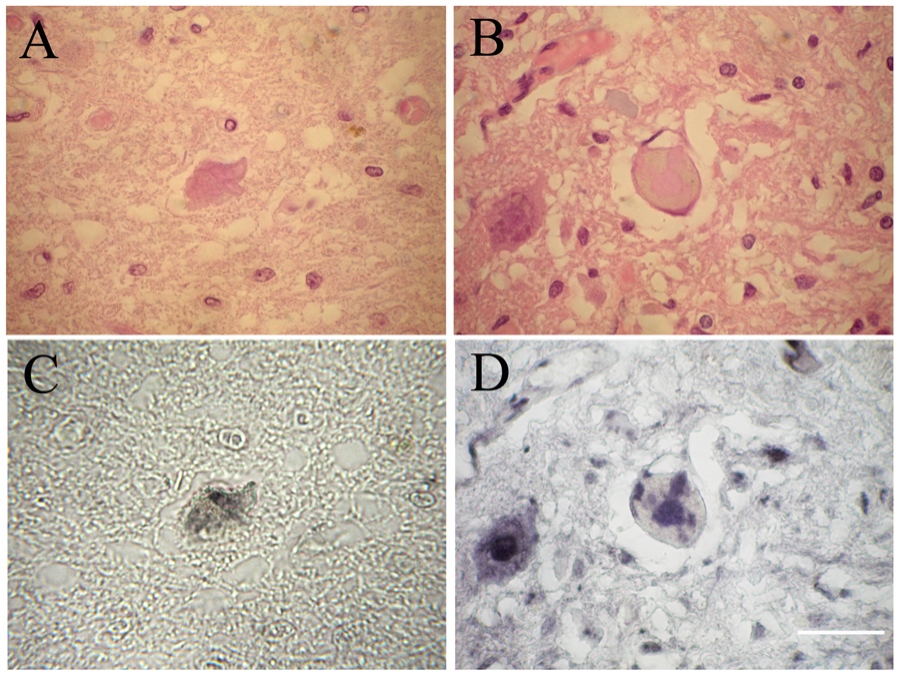
LBHIs immunopositive for 14-3-3β or 14-3-3γ in FALS patients. A and B are the same sections as C and D, respectively. The identical LBHIs observed on H&E (A, B) in the anterior horn cells are intensely immunostained with 14-3-3β (C) and 14-3-3γ (D). Bar indicates 50 µm.

In all the three FALS cases, LBHIs were observed inside the anterior horn cells (Figure 1A–C). All the LBHIs observed on H&E showed strong pan 14-3-3 immunoreactivity (Figure 1D–F). Using 14-3-3 isoform-specific antibodies, all the LBHIs detected by H&E (Figure 2A, B) were intensely immunopositive both for 14-3-3β (Figure 2C) and 14-3-3γ (Figure 2D).

**Figure 3:**
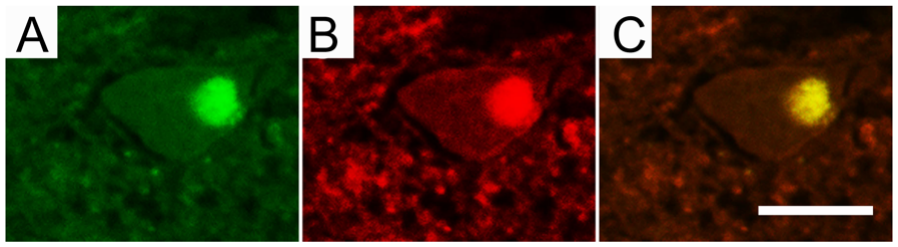
A LBHI double-positive for 14-3-3 and SOD1 in a FALS patient. A LBHI in an anterior horn cell is immunostained for pan 14-3-3 (A, green) and SOD1 (B, red), and the merged image is shown in C (yellow). Bar indicates 50 µm.

Double immunofluorescent-stained sections showed that pan 14-3-3 was co-localized with SOD1 in the LBHI (Figure 3).

### Mutant SOD1 immunoreactivy in the mutant SOD1-Tg, the wild-type SOD1-Tg, and non-Tg wild-type mice

**Figure 4:**
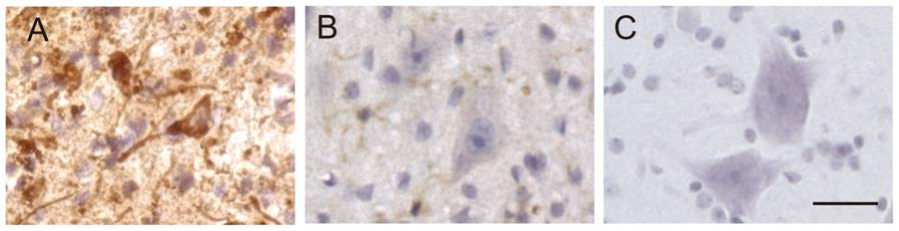
Neuronal inclusions immunopositive for C4F6 in mice. Strong immunoreactivity for C4F6 (A) was observed in the somatodendritic compartment with cytoplasmic inclusions in the mutant SOD1-Tg mice. Immunoreactivity for C4F6 was restricted to glial cells morphologically consistent with microglia in the wild-type SOD1-Tg mice (B) and absent in the non-Tg wild-type mice (C). Bar indicates 50 µm.

In mutant SOD1-Tg mice, C4F6 immunoreactivity was observed in the remaining anterior horn cells with cytoplasmic inclusions (Figure 4A). Immunoreactivity for C4F6 was restricted to the glial cells that were morphologically consistent with microglia in wild-type SOD1-Tg mice (Figure 4B) and absent in non-Tg wild-type mice (Figure 4C).

### 14-3-3 immunoreactivity in the mutant SOD1-Tg, the wild-type SOD1-Tg, and non-Tg wild-type mice

**Figure 5:**
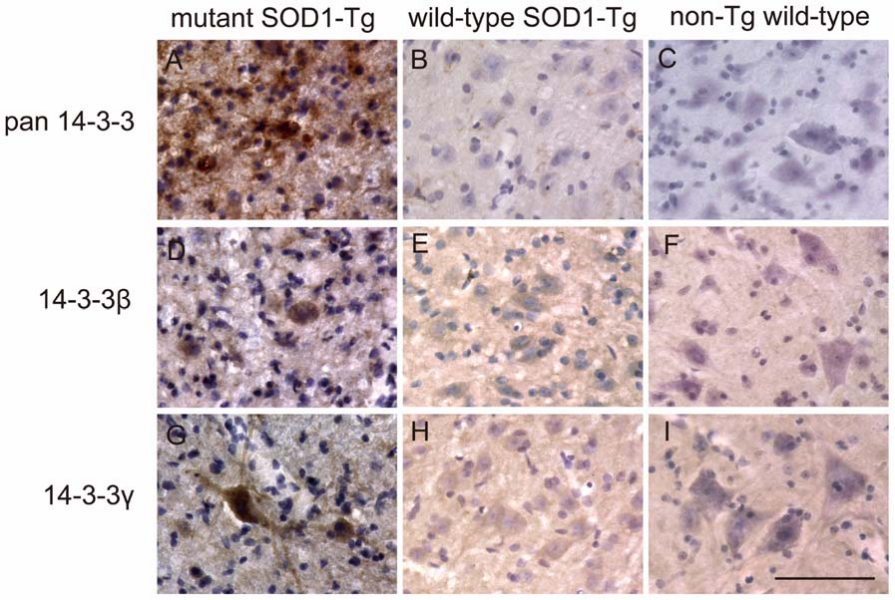
Neuronal inclusions immunopositive for pan 14-3-3, 14-3-3β, and 14-3-3γ in the spinal cord of SOD1-Tg mice. In mutant SOD1-Tg mice, strong immunoreactivity for pan 14-3-3 (A), 14-3-3β (D), and 14-3-3γ (G) were observed in the neuronal cytoplasm or neuronal process of the lumbar anterior horn cells. Such immunoreactivities were not observed in the wild-type SOD1-Tg (B, E, H) or non-Tg wild-type mice (C, F, I). Bar indicates 50 µm.

Pan 14-3-3, 14-3-3β and 14-3-3γ immunoreactivities were grossly different between the mutant SOD1-Tg and the wild-type SOD1-Tg or non-Tg wild-type mice (Figure 5).

Such 14-3-3 immunoreactivities were strong in most of the remaining anterior horn cells of the mutant SOD1-Tg mice (Figure 5A, D, G), although they were not observed in the wild-type SOD1-Tg (Figure 5B, E, H) or non-Tg wild-type mice (Figure 5C, F, I).

### Double immunofluorescent staining of C4F6 and 14-3-3β or 14-3-3γ in SOD1-Tg mice

As described above, the strong immunoreactivity of 14-3-3β and 14-3-3γ were observed in the mutant SOD1-Tg mice but not in the wild-type SOD1-Tg mice. Wherein, the distribution of immunoreactivity for C4F6, 14-3-3β and 14-3-3γ was analyzed.

**Figure 6:**
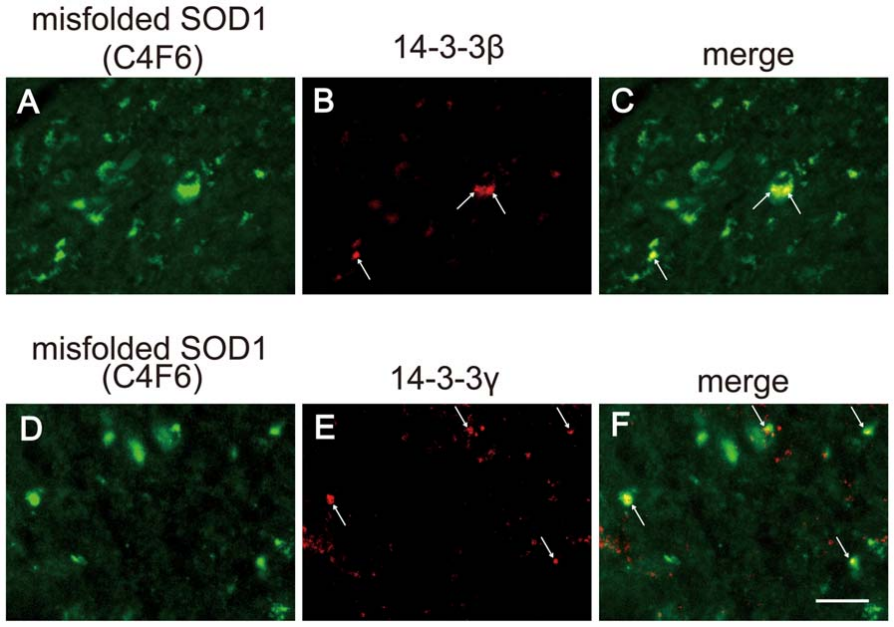
Neuronal inclusions double-positive for C4F6 and 14-3-3 in the spinal cord of mutant SOD1-Tg mice. Immunofluorescence for C4F6 (A, D, green), 14-3-3β (B, red), and 14-3-3γ (E, red), double immunofluorescence for C4F6 and 14-3-3β (C, merge), and double immunofluorescence for C4F6 and 14-3-3γ (F, merge) are shown in the anterior horn cells. Bar indicates 20 µm.

All the three immunoreactivities were observed in the neuronal somata of the anterior horn cells. Furthermore, double immunofluorescent staining showed that both 14-3-3β and 14-3-3γ were partially co-localized with C4F6 in mutant SOD1-Tg mice (Figure 6).

In negative immunohistochemical controls, some sections were incubated with the primary antibody (0.2 µg/ml) preabsorbed with an excess amount of the antigenic peptides, pan 14-3-3, 14-3-3β and 14-3-3γ (10 µg/ml). No specific immunopositive staining was detected in these control sections.

### Other 14-3-3 isoforms in the mutant SOD1-Tg mice and the wild-type SOD1-Tg mice

Immunoreactivitiy for 14-3-3ε, η, θ, σ, and ζ was observed in the neuronal somata and processes in the spinal cord. However, there were no remarkable differences in the distribution or intensity of the immunoreactivities between the mutant SOD1-Tg and wild-type SOD1-Tg mice.

## Discussion

In this study, LBHIs in all FALS cases showed intense pan 14-3-3, 14-3-3β and 14-3-3γ immunoreactivities. Furthermore, the double immunofluorescent study showed 14-3-3 proteins were co-localized with SOD1 in LBHIs. Such distribution patterns were quite similar to those of the mutant SOD1-Tg mice. This is the first report that demonstrates a close relationship between 14-3-3 and SOD1 both in patients with FALS and mutant SOD1-Tg mice.

We have previously reported the localization of 14-3-3 proteins in the ubiquitinated intraneuronal inclusions in the anterior horn cells from patients with sporadic ALS [12]. We also already reported 14-3-3 immunoreactivity in the LBHIs in the anterior horn cells from a patient with FALS, with a two-base pair deletion in the SOD1 gene [19]; however, the co-localization of SOD1 and 14-3-3 was not assessed. Therefore the role of 14-3-3 proteins in LBHI formation with a SOD1 mutation has remained unclear. The co-localization of 14-3-3 and SOD1 in the LBHIs in both FALS patients and mutant SOD1-Tg mice suggested that 14-3-3 may play an important role in the formation of SOD1-containing LBHIs. The similar 14-3-3 positivity in the LBHI of sporadic ALS and FALS with SOD1 mutation further suggests that 14-3-3 is involved in the pathogenesis of ALS, irrespective of whether it is sporadic or familial.

Among the various isoforms of 14-3-3 protein, Kaneko and Hachiya proposed the possibility that a distinctive function of 14-3-3ζ might be as a sweeper for misfolded proteins, such as aggregates or inclusion bodies [20]. Santpere et al. suggested that the 14-3-3γ and 14-3-3ζ isoforms may be the targets of oxidative damage in Alzheimer's disease [21], and some neurofibrillary tangles were reported to be immunolabeled with 14-3-3β and 14-3-3γ [22]. Similarly, 14-3-3 proteins have been co-localized in Lewy bodies [23] and in glial cytoplasmic inclusions from patients with multiple system atrophy [24]. In our recent study, 14-3-3β and 14-3-3γ were strongly expressed in the neuronal somata and processes of anterior horn cells in the spinal cord of mutant human α-synuclein (A53T)-Tg mice, an animal model of Parkinson's disease (PD) [25]. Therefore, 14-3-3β and 14-3-3γ may be the key isoforms associated with the formation of α-synuclein- and SOD1-containing inclusions. This raises the possibility that there might be a common mechanism for inclusion formation at least between ALS and PD.

An insufficient function of the molecular chaperones may be directly involved in the loss of motor neurons in ALS [26], [27]. Under non-pathological conditions, 14-3-3 proteins play important roles in signal transduction, apoptotic cell death and cell cycle control. 14-3-3 proteins inhibit apoptosis by binding to and inactivating pro-apoptotic proteins, including the mitochondrial Bcl-2 family member BAD, apoptosis signal-regulating kinase 1 (ASK1), and the Forkhead transcription factor FKHRL1 [10]. Therefore, the sequestration of 14-3-3 may cause neuronal dysfunction and thus contribute to cell death. Strong immunoreactivity for 14-3-3 in the LBHIs of FALS patients and in the mutant SOD1-Tg mice suggested that 14-3-3 proteins are trapped in the LBHIs, and this deficiency of the 14-3-3 proteins causes motor neuronal death in patient with FALS.
